# Meta-analysis of the effects of drug-coated balloons among patients with small-vessel coronary artery disease

**DOI:** 10.1097/MD.0000000000015797

**Published:** 2019-05-31

**Authors:** Jing-qi Yang, Jin-hua Peng, Ting Xu, Li-yun Liu, Jie-hong Tu, Shun-hui Li, Hui Chen

**Affiliations:** a Department of Cardiovascular Medicine, The Third Affiliated Hospital of Nanchang University, The First Hospital of Nanchang, Nanchang City, Jiangxi Province, China; b Women and Child Health Care Hospital of Jiangxi Province, China.

**Keywords:** bare-metal stents, drug-coated balloon, drug-eluting stents, small-vessel coronary artery disease, systematic review

## Abstract

**Objective:**

This study evaluated the clinical value of drug-coated balloons for patients with small-vessel coronary artery disease (SVD).

**Methods:**

A computerized literature search was performed using the databases to conduct a meta-analysis and evaluate the clinical value of drug-coated balloons among patients with SVD.

**Results:**

This review enrolling 1545 patients receiving drug-coated balloons and 1010 patients receiving stents (including drug-eluting stents and bare-metal stents). The meta-analysis results showed that the incidence of major adverse cardiovascular events among patients with SVD did not significantly differ between the drug-coated balloon group and the stent group within 1 postoperative year (odds ratio = 0.81, *P* = .5). A subgroup analysis showed that the incidence of myocardial infarction among the drug-coated balloon group was significantly lower than that among the stent group (odds ratio = 0.58, *P* = .04). Nevertheless, the late lumen loss of the drug-coated balloon group was significantly lower than that of the stent group (mean difference = 0.31, *P* = .01).

**Conclusions:**

Drug-coated balloons can be used to effectively reduce the incidence of myocardial infarction in patients with SVD within 1 year and decrease the extent of late lumen loss without increasing the incidence of major adverse cardiovascular events.

## 1 Introduction

According to the latest epidemiological studies, the prevalence and mortality of cardiovascular diseases continue to increase. According to statistics, approximately 11 million patients have coronary heart disease.^[1]^ Percutaneous coronary intervention (PCI) is the major treatment method for coronary heart disease.^[2]^ Small-vessel disease (SVD) has been found in the coronary arteries of patients with coronary heart disease on coronary angiography, especially in those with concomitant diabetes. SVD generally refers to lesions in blood vessels <2.8 mm in diameter. For patients with SVD in their coronary arteries, stent implantation is associated with high incidences of small-vessel rupture, dissection, and vascular restenosis. Granada et al^[3]^ used a paclitaxel-coated balloon in an animal model and found that when the balloon was dilated, the paclitaxel on the balloon's surface rapidly penetrated into the vessel wall. Although most of the paclitaxel was washed away from the vessel wall by the arterial blood, some remained on the surface of the vessel wall and slowly penetrated into the arterial tissue, thereby inhibiting the proliferation of smooth muscle cells. Initially, drug-coated balloons were widely used clinically for patients with in-stent restenosis. However, DES in small vessels are still associated with a relatively high incidence of restenosis.^[4]^ In the Cortese's^[5]^ trial, the drug-coated balloons (DCB) group failed to show equivalence to drug-eluting stents (DES) regarding angiographic end points during PCI of small coronary arteries, and the primary end point was not met. Recently, the Balloon Elution and Late Loss Optimization (BELLO) randomized trial demonstrated that the treatment of DVD with a paclitaxel DCB was associated with less angiographic late loss and similar rates of restenosis and revascularization when compared with paclitaxel-eluting stents at 6 months and a lower incidence of major adverse cardiovascular event (MACE) at 3-year follow-up.^[6]^ Nevertheless, the clinical efficacy of drug-coated balloons and their long-term prognoses remain controversial with regard to patients with SVD. In this study, a meta-analysis of the clinical application of drug-coated balloons among patients with SVD was performed to explore their clinical application among these patients and to provide a new clinical treatment for SVD.

## 2 Materials and methods

### 2.1 Retrieval strategy

A computerized literature search was conducted to collect information from the PubMed, EMBASE, and Cochrane Library databases. The retrieval period ranged from the creation date of each database to December 2018. The keywords for retrieval included SVD OR small vessel lesions, Coronary heart disease OR CHD, and drug-coated balloon OR paclitaxel-eluting balloon. According to literature retrieval requirements, different retrieval strategies were developed for different databases, and relevant articles concerning the application of drug-coated balloons among patients with SVD were collected.

### 2.2 Inclusion criteria

The included studies met the following criteria: all studies were randomized controlled trials (RCTs), cohort studies, case–control studies, or one-arm studies; all participants were patients with coronary heart disease whose clinical coronary angiography results suggested major vessel diameters <2.8 mm; the observation indicators included types of DCB and drug DES or bare-metal stents (BMS), applicable populations, and definitions of SVD; the outcome indices included major cardiovascular adverse event, death, myocardial infarction (MI), target lesion revascularization (TLR), target vessel revascularization (TVR), and late lumen loss (LLL), which were monitored to evaluate the outcomes or prognoses of patients who received DCB or stents (including DES and BMS); and the literature publication period ranged from the creation date of each database to December 2018, and the research development time was not limited. The sample size of the original research was not limited.

### 2.3 Exclusion criteria

Studies that satisfied any of the following criteria were excluded: duplicate publications; studies that did not follow up with the outcome indices or discuss the prognoses of patients after the application of DCB; studies with only abstracts or conference proceeding available (i.e., incomplete information provided); studies with samples of patients without SVD; reviews, commentaries, expert reviews, or studies with animal/basic research experiments; studies of critically ill patients with SVD with surgical diseases or other noncardiovascular diseases; and clinical studies with an attrition rate >20%.

### 2.4 Screening of selected studies

Two researchers served as independent evaluators by retrieving the titles and abstracts of the relevant literature in strict accordance with the inclusion and exclusion criteria. After removing unsatisfactory documents, the literature that was eligible for the study was read thoroughly, and the data from the selected literature were retrieved. If the 2 researchers had conflicting opinions with regard to a study, then the literature was either included or excluded depending on the results of their discussion result or based on the opinion of the article's corresponding author. After the 2 researchers cross-checked the extracted data, they contacted the corresponding authors to obtain any missing information.

### 2.5 Evaluation of document quality and data retrieval

The Newcastle-Ottawa Scale (NOS)^[7]^ was used to evaluate the quality of RCTs and cohort studies for the meta-analysis. The studies were scored using a scale that contained 3 aspects of participant selection: comparability, outcomes, and exposures. The maximum score of the scale is 9, and scores of 5 to 9 indicate high quality. The quality of one-arm studies was assessed using MINORS entries^[8]^ with 8 evaluation indicators. Each indicator was scored from 0 to 2 points, and a total score >8 points suggests a high-quality study.

General information was retrieved from the studies, including the first author, time, type of research design (prospective or retrospective), diagnostic methods, and number of patients. The prognostic indicators of patients with SVD were also extracted, including the rates of MACEs, death, MI, TLR, TVR, and LLL.

### 2.6 Ethical review

This is a meta-analysis article, and the statistical analysis of large collection of analysis results from individual studies for the purpose of integrating the findings does not involve ethical review.

### 2.7 Statistical methods

Data processing was performed using Cochrane Collaboration-specific software (RevMan 5.0). Count data are expressed as odds ratios (ORs) and 95% confidence intervals (CIs). The heterogeneity among studies was tested using the χ^2^ test. Values of *P* > .1 and *I*^2^ <50% indicated no significant difference in study heterogeneity. In the event of study homogeneity, a fixed-effect model was used for the combined analysis; if heterogeneity was present, then subgroup and sensitivity analyses were employed to detect the causes of clinical and statistical heterogeneity. If heterogeneity was still present after interference from the above factors was excluded, then a random-effects model was used for the combined analysis. Sensitivity analysis was carried out by an exclusion method whereby each study was excluded one by one and a new analysis was carried out each time. Egger's tests were performed using Stata 12.0 to evaluate publication bias, with a *P* < .05 corresponding to positive evidence of publication bias.

## 3 Results

### 3.1 Studies included and patients’ characteristics

**Figure 1:**
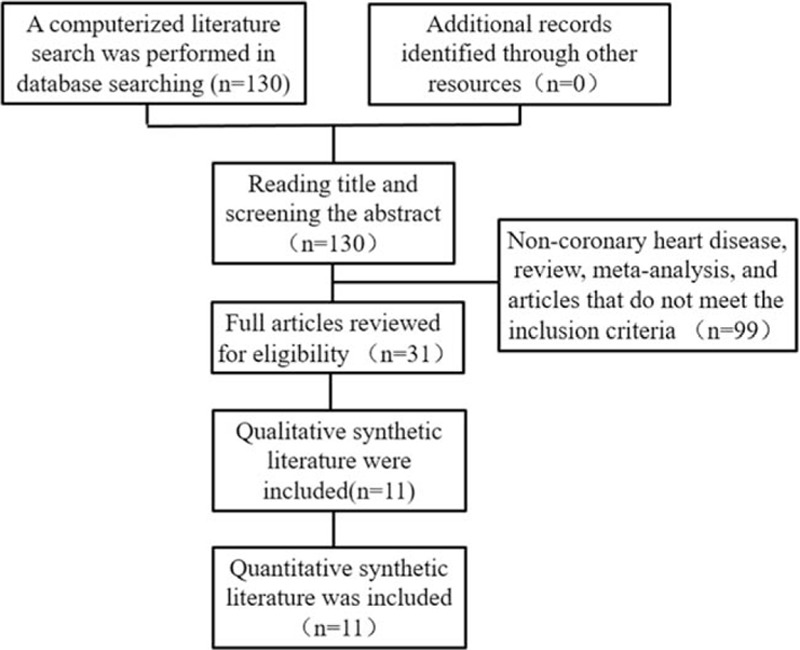
Search strategy and literature screening process and results.

The results were searched separately in each database according to the proposed search terms, and 130 initial search resulted were retrieved. After screening by inclusion criteria and exclusion criteria, a total of 3 single-arm studies^[9–11]^ and 8 case–control studies^[5,6,12–17]^ were included, enrolling 1545 patients receiving DCB and 1010 patients receiving DES or BMS (Fig. 1). The basic clinical data of the literature are shown in Table 1. According to the MINORS item and the NOS scale, the quality of the 11 articles included were evaluated, and the included articles were all of good quality.

**Table 1:**
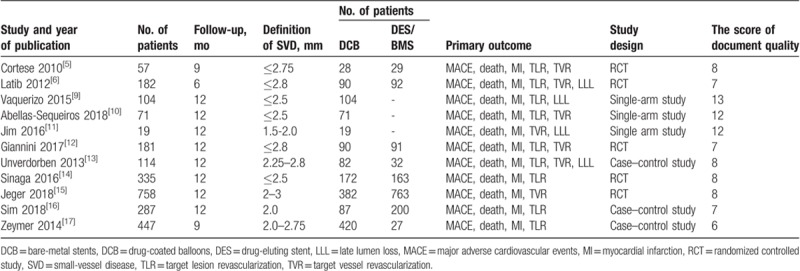
Baseline demographic and clinical characteristics.

### 3.2 Incidence of MACEs in patients with SVD receiving DCB within 1 postoperative year

**Figure 2:**
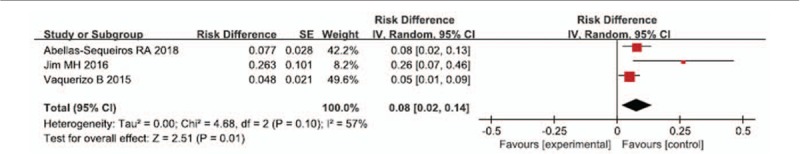
Summary incidence of MACE events in patients with SVD receiving drug-coated balloons.

Three single-arm studies were included for the combined effect size analysis of the incidence of MACEs. Because of the heterogeneity, the random-effects model was used in the analysis. The results showed that the risk of MACE in the SVD patient receiving DCB within 1 postoperative year was 0.08 [95% CIs=(0.02–0.14), *P* < .05] (Fig. 2).

### 3.3 Primary outcomes of long-term prognosis in patients with SVD receiving DCB and DES/BMS

**Figure 3:**
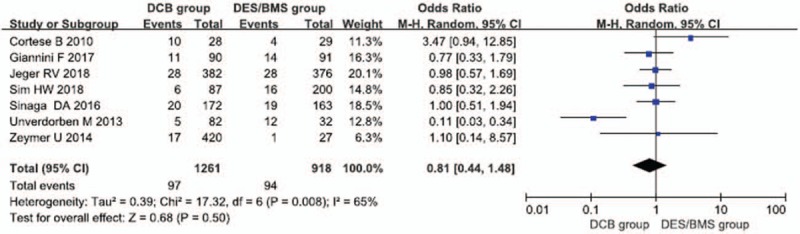
Forest map with long-term prognosis in patients with SVD receiving drug-coated balloons and drug-eluting stents.

**Figure 4:**
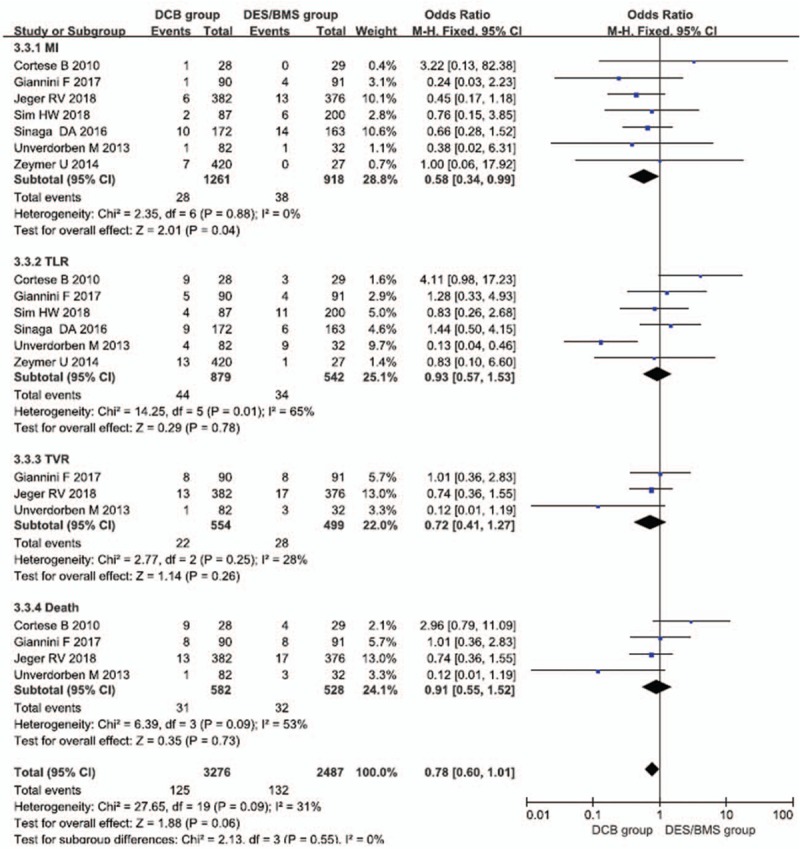
Subgroup analysis of long-term prognosis in patients with SVD receiving drug-coated balloons and drug-eluting stents. MI = myocardial infarction, TLR = target lesion revascularization, TVR = target vessel revascularization.

Seven case–control studies were reported the primary outcome of MACE. The meta-analysis results showed that the incidence of MACEs among patients with SVD did not significantly differ between the DCB group and the DES/BMS group within 1 postoperative year (OR = 0.81, 95% CIs = [0.44–1.48], *P* = .5). However, the subgroup analysis showed that the incidence of MI among the DCB group was significantly lower than that among the DES/BMS group (OR = 0.58, 95% CIs = [0.34–0.99], *P* = .04); the incidences of target lesion revascularization (OR = 0.93, 95% CIs = [0.57–1.53], *P* = .78), target vessel revascularization (OR = 0.72, 95% CIs = [0.41–1.27], *P* = .26), and death (OR = 0.91, 95% CIs = [0.55–1.52], *P* = .73) did not significantly differ between the 2 groups (Figs. 3 and 4).

### 3.4 Secondary outcomes

**Figure 5:**
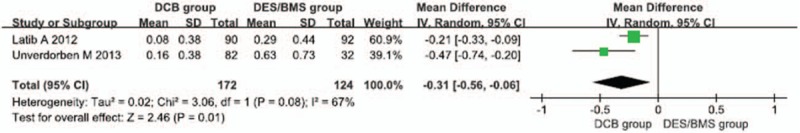
Forest map of the risk of late lumen loss in SVD patients receiving drug-coated balloons and drug-eluting stents after 6 months.

Although only 2 articles ^[6,13]^ analyzed the incidence of LLL in SVD patients receiving DCB and DES/BMS after 6 months, the results show compared with the stent group, the DCB group was associated with lower incidences of the LLL (mean difference  = 0.31, 95% CIs = [0.06–0.56], *P* = .01) (Fig. 5).

### 3.5 Sensitivity analysis and publication bias

To find the influence of an individual study on the pooled ORs, we excluded one study each time and investigated if there was any change of pooled ORs and heterogeneity. In the primary result, we found that when we excluded the result of study of Unverdorben et al^[13]^ the heterogeneity decreased dramatically (*I*^2^ dropped from 67% to 0%). The most important reason was that the stent group was treated by BMS, which may cause a higher risk of restenosis. However, similar results were shown with the random-effects model (OR = 1.03, 95% CI = 0.74–1.03). Therefore, the initial result of this comparison was regarded as reliable. There was no evidence of publication bias determined by Begg's (*P* = .76) and Egger's tests (*P* = .81) in any of the analyses.

## 4 Discussion

PCI has been widely promoted for patients with coronary heart disease. According to statistics, 3.6 million patients with coronary heart disease underwent coronary intervention in 2015.^[18]^ With the increasing application of PCI, the problem of in-stent restenosis is increasing. Drug-coated balloons are now widely used for patients with in-stent restenosis.^[19]^ However, the application of DCB for patients with SVD remains controversial because different studies have obtained conflicting conclusions. In theory, a balloon coated with a paclitaxel-based drug should inhibit the intimal hyperplasia after it contacts the vessel wall without leaving a metal mesh in the blood vessel, thereby reducing the inflammation of the intima. Theoretically, it can achieve better clinical benefits than drug-eluting stents or simple balloon dilation.^[20]^ However, clinical studies have found that the clinical benefit of drug-coated balloons is not inferior to drug-eluting stents or bare-metal stents.

The incidence of SVD among patients with coronary artery disease is high, especially among those with diabetes. Women and smaller patients also have small coronary vessels. Small-vessel lesions are mostly located in the distal and medium segments of the coronary artery, and these lesions are complex, often combined with diffuse and twisted lesions. In current RCTs, drug-coated balloons have been shown to reduce the incidence of long-term LLL in patients with SVD; furthermore, MACEs are rare, which indicates that the application of DCB shows good prospects for patients with SVD.

Several clinical databases were searched in this study, and 3 single-arm studies and 8 case–control studies were collected that included patients receiving 1545 DCB and 1010 patients receiving DES or BMS. The included studies were systematically evaluated. In the 3 one-arm studies, the overall incidence of MACEs among patients with SVD was 8% within 1 year after drug-coated balloon therapy. In the 8 case–control studies included, the incidences of MACEs, TLR, TVR, and death did not significantly differ between patients with SVD receiving DCB therapy and those receiving DES/BMS stent therapy within 12 months (*P* > .05). However, the risks of MI and LLL in the DCB group were significantly lower than those in the DES/BMS group (*P* < .05). DCB can be effectively and safely applied to patients with SVD. At present, most of the drug-coated balloon's surface is coated by a paclitaxel-based drug. Paclitaxel has satisfactory fat solubility, inhibits endothelial proliferation, and effectively slows cell proliferation. However, after the DCB is dilated in patients with SVD, the balloon comes in full contact with the vessel wall, and paclitaxel is rapidly taken up by the vascular wall tissue. Paclitaxel can inhibit the proliferation of vascular smooth muscle cells within 14 days and inhibit intimal hyperplasia 4 weeks after treatment, thereby effectively reducing the occurrence of MACEs.^[21]^ After the implantation of DES or BMS in patients with SVD, the risks of small blood vessel rupture and long-term restenosis are high.

The main attraction of DCB use in the patients with SVD is that there is no foreign body implanted, thus reducing the risk of a late inflammatory response to device components. We also confirm the result that the risks of LLL in the DCB group were significantly lower than those in the DES/BMS group. DCB may be particularly advantageous over DES/BMS in the treatment of SVD by providing an immediate and homogenous drug uptake, avoiding inflammatory reaction to stent struts or polymers, and respecting the normal vessel anatomy. Furthermore, in the structure and function, the balloon is coated with the antimitotic drug paclitaxel to reduce restenosis and has the benefit of a matrix of paclitaxel and a hydrophilic spacer on its surface, which creates a large contact surface area between the lipophilic drug and the vessel wall and increases the bioavailability of the embedded drug and facilitates its rapid drug absorption by the vessel wall. This use of a hydrophilic spacer allows the uniform and complete administration of the drug after first balloon inflation.^[22,23]^

Seven of the case–control studies included in this paper were of high quality, but 2 studies had 9-month follow-up periods, whereas the remaining studies had 12-month follow-up periods. The difference in events might also produce heterogeneity. However, after the analysis of the combined effect size of the specific cardiovascular adverse events included as MACEs, we found that this heterogeneity might be derived from the risk of TLR in both groups (*I*^2^ = 58%). Therefore, the combined effect size of the results of the 7 articles was analyzed. Heterogeneity was found among the 3 single-arm studies. However, if the analysis was performed excluding Jim et al,^[8]^ then the heterogeneity was 0; thus, the heterogeneity was primarily due to that study. In addition, of the included studies, factors such as different threshold values for small blood vessels and disparate observation indicators for patient prognoses might cause heterogeneity. Moreover, the geographical and sample size differences among studies will also result in high heterogeneity.

A meta-analysis is a statistical method used to combine multiple independent studies in a systematic review. This study also has drawbacks. The papers included in this article were case–control studies, but their follow-up durations differed. If multiple long-term follow-up assessments are conducted, then the medical evidence will be more sufficient. Different levels of heterogeneity and bias will also affect the research results. The sample size differences among the included studies is large; thus, bias tends to occur.

In summary, this meta-analysis found that drug-coated balloons effectively reduce the incidences of long-term LLL in patients with SVD without increasing the number of MACEs. Therefore, this study provides a new clinical intervention strategy for patients with SVD and a better treatment basis to improve the prognosis of these patients.

## Author contributions

J-qY and J-hP designed the meta-analysis; J-qY and TX carried out the meta-analysis; L-yL and J-hT performed the data analyses; S-hL provided the funding and revised the manuscript; J-qY and HC wrote the manuscript.

**Conceptualization:** Jin-hua Peng.

**Data curation:** Jing-qi Yang.

**Funding acquisition:** Shun-hui Li.

**Methodology:** Ting Xu, Li-yun Liu.

**Software:** Li-yun Liu, Jie-hong Tu.

**Writing – original draft:** Jing-qi Yang, Hui Chen.

**Writing – review and editing:** Shun-hui Li, Hui Chen.
